# Effects of ractopamine hydrochloride on nutrient digestibility and nitrogen excretion of finishing beef cattle

**DOI:** 10.1093/tas/txab036

**Published:** 2021-03-07

**Authors:** Bailey N Harsh, Brady J Klatt, Mareah J Volk, Angela R Green-Miller, Joshua C McCann

**Affiliations:** University of Illinois at Urbana-Champaign, IL 61801, USA

**Keywords:** beta-agonist, digestibility, feedlot cattle, ractopamine hydrochloride

## Abstract

The objective was to quantify the effects of the beta-adrenergic agonist (β-AA) ractopamine hydrochloride (Actogain, Zoetis, Parsippany, NJ) on nitrogen excretion and nutrient digestibility in feedlot cattle. In experiment 1, 12 Simmental × Angus steers were blocked by bodyweight (531 ± 16 kg) and used in a randomized complete block design. Dietary treatments included: 1) a control without β-AA (CON) or 2) 400 mg/steer/d ractopamine hydrochloride (RAC) for 35 d before slaughter. Diets contained (DM basis) 55% dry-rolled corn, 20% corn silage, 15% modified wet distillers grains with soluble, and 10% supplement. For each block, total collection of feed, orts, feces, and urine were conducted for two 5 d sampling periods during week 2 and 4 of RAC supplementation. No interaction (*P* > 0.21) between treatment and collection period was observed for any parameter evaluated. Dietary treatment had no effect (*P* = 0.51) on DMI, but RAC had decreased fecal DM output (*P* = 0.04) compared with CON. Thus, RAC had greater apparent total tract DM digestibility (77.2 vs. 73.5%; *P* < 0.01), *N* digestibility (72.4 vs. 69.4%; *P* = 0.01), and NDF digestibility (65.6 vs. 60.2%; *P* < 0.01) than CON. Although treatment did not affect nitrogen intake (*P* = 0.52), RAC tended to reduce total nitrogen excretion (113.3 vs. 126.7 g/d; *P* = 0.10) compared with CON due to a tendency for decreased fecal nitrogen output (53.9 vs. 61.3 g/d; *P* = 0.10). However, dietary treatment had no effect (*P* = 0.53) on urinary nitrogen output or percentage of urinary nitrogen excreted as urea (*P* = 0.28). Experiment 2 was an in vitro experiment conducted to validate the effects of RAC on nutrient digestibility using Simmental × Angus heifers (451 ± 50 kg). Rumen fluid was collected individually by stomach tube from CON- (*n* = 9) and RAC-fed (*n* = 10) heifers to inoculate bottles containing a CON or RAC-containing substrate in a split–plot design. No interaction between rumen fluid source and in vitro substrate was observed. Greater IVDMD (*P* = 0.01) was observed in rumen fluid from RAC-fed heifers compared with rumen fluid from CON-fed heifers. The inclusion of RAC in the in vitro substrate increased IVDMD (*P* < 0.01). Overall, feeding RAC increased microbial digestion of the dry-rolled corn-based finishing diet to increase total tract dry mater digestion by 5% and reduce nitrogen excretion by 10.6% in the 35 d period prior to slaughter.

## INTRODUCTION

Improving the environmental sustainability of beef production has come under increasing producer, consumer, and regulatory scrutiny (Johnson and Johnson, 1995; Capper, 2011). Feedlot cattle production systems are considered a major source of excess nitrogen excretion in the environment (Schröder et al., 2005). Ammonia and methane emissions, nutrient runoff, nitrate leaching, and soil denitrification have surfaced as primary targets in the efforts to mitigate and minimize the environmental effects of intensive beef production systems (Hristov et al., 2011; Prados et al., 2016). Indications for beta-adrenergic agonists (β-AA) such as ractopamine hydrochloride (RAC; Actogain, Zoetis, Parsippany, NJ) and other growth-promoting technologies (GPT) include improvements in efficiency of BW gain and carcass leanness. Furthermore, GPT have enabled producers to use fewer land and feed resources while reducing manure output and greenhouse gas emissions compared with steers raised without GPT (Cooprider et al., 2011; Capper and Hayes, 2012). Despite these secondary improvements in environmental sustainability associated with the use of β-AA, relatively little is known about the direct effects of β-AA on nitrogen excretion, methane emissions, and nutrient digestibility. Beta-agonists are proven to increase carcass protein concentration by up to 8% in finished steers, improving retail cut yields (Boler et al., 2009; Hilton et al., 2009). If β-AA can increase carcass protein accretion, then nitrogen retention should be increased, thereby reducing nitrogen excretion into the environment. However, the efficaciousness of β-AA is known to decrease over time because of receptor desensitization (Hausdorff et al., 1990). Therefore, the objective was to quantify the effects of RAC and period of RAC inclusion (d 8–13 vs d 22–27) on nitrogen excretion and nutrient digestibility through a pair of in vivo and in vitro experiments.

## MATERIALS AND METHODS

Animal procedures were approved by the University of Illinois Institute of Animal Care and Use Committee (IACUC 14278) and followed the guidelines recommended in the Guide for the Care and Use of Agricultural Animals in Agricultural Research and Teaching (FASS, 2010).

### Experiment 1: Nitrogen Excretion and Nutrient Digestibility

Twelve Simmental × Angus crossbred steers were used in a randomized complete block design to evaluate the effects of ractopamine hydrochloride (Actogain, Zoetis, Parsippany, NJ). Dietary treatments were blended with 0.45 kg ground corn per steer and top-dressed on diets daily and included: 1) a control without ractopamine hydrochloride (CON) or 2) 400 mg/steer/d ractopamine hydrochloride (RAC) for 35 d before slaughter. Steers receiving only a single implant after weaning containing 80 mg trenbolone acetate and 16 mg estradiol (Component TE-IS; Elanco Animal Health, Greenfield, IN) were used, to isolate observations of the potential treatment effects. Steers were split into a heavy (initial BW = 534 ± 22 kg) and light (initial BW = 529 ± 8 kg) block by weight and metabolism trial procedures were conducted sequentially beginning with the heavy block and 4 weeks later with the light block. All steers were fed in separate pens for 1 week to determine individual feed intake and acclimated to metabolism stalls prior to trial initiation.

The basal diet contained 55% dry-rolled corn, 20% corn silage, 15% modified wet distillers grains with solubles, 10% supplement (DM basis, Table 1), and was formulated to meet or exceed National Academies of Sciences, Engineering, and Medicine (NASEM, 2016) recommendations. Diets were fed once daily with the amount of feed offered continually adjusted throughout the experiment to ensure ad libitum intake and complete ingestion of ractopamine while minimizing feed wastage. Water was available on an ad libitum basis. Steers were housed in metabolism tie stalls at the University of Illinois Beef Cattle and Sheep Field Research Laboratory in Urbana, IL. Stalls (2.3 × 1.3 m) were equipped with individual feed bunks and non-siphoning, automatic water bowls. Stalls also contained the Ruminant Emission Monitoring System (REMS) for determination of eructated gas emissions (Maia et al., 2015b). The barn was equipped with heat, ventilation, and air-conditioning systems, providing a controlled environment (18.3°C) for steers on trial.

**Table 1.** Diet and nutrient composition of feedlot diet in Exp. 1

Ingredient	Inclusion, %DM
Dry-rolled corn	55
Corn silage	20
MWDGS	15
Supplement^*^	10
Analyzed nutrient content, % DM	
DM	54.0
CP	14.1
NDF	25.4
Ether extract	4.2

^*^Supplement contained: 74.4% ground corn, 17.1% limestone, 6.5% urea, 0.98% trace mineral, and vitamin premix (8.5% Ca, 5% Mg, 7.6% K, 6.7% Cl, 10% S, 0.5% Cu, 2% Fe, 3% Mn, 3% Zn, 278 mg/kg Co, 250 mg/kg I, 150 mg/kg Se, 2.205 KIU/kg Vit A, 662.5 KIU/kg Vit D, 22,047.5 IU/kg Vit E.) 0.81% liquid fat, 0.17% Rumensin 90 (Elanco Animal Health, Greenfield, IN), 0.11% Tylan 40 (Elanco Animal Health, Greenfield, IN).

#### Sample collection.

For each block, two-5 d collection periods were conducted on d 8–13 and d 22–27 with a one-week rest period between collections. Additionally, a one-week treatment adaptation period was observed prior to the first collection of each block to allow steers to acclimate to feeding in metabolism stalls. Control or RAC diets were fed continuously during the 35 d period including adaptation and rest weeks. Feed samples were collected for each day of the collection period (d 1–5). Orts were weighed to determine daily feed intake and were sampled as well. Feed and ort samples were stored at –20°C until analysis.

On the second day of each collection period, fecal bags were attached to each steer to determine fecal output for the entire 5 d period. Feces were collected in waterproof canvas bags connected to the steer by a leather harness, removed from fecal bags twice daily, and weighed at 0600 h the following morning. A 5% subsample of the total daily fecal weight was added to a composite sample for the entire collection period. Fecal composite samples were stored at –20°C until analysis.

Urine collection funnels were attached to steers on d 1 of the collection period and used to collect urine for the entire 5 d period. Silicone urine funnels were positioned around the steer’s sheath. Continuous vacuum suction was applied to the funnel system to aid in the collection of urine into 18.9 L plastic collection vessels via plastic hoses. During the 5 d of urine collection, steers were observed to ensure funnels and collection system remained in place and collections accurately represented total urine output. On d 1, urine was collected without acidification in containers placed on ice for analysis of nitrogen species. During the remaining days of urine collection, urine was acidified by adding 175 mL of 6 N HCl in urine containers. Urine was weighed twice daily at 0600 and 1800 h and a 2% subsample of each weigh periods urine output was added to a composite sample representative of the entire collection period. Urine was stored at –20°C.

#### Eructation analysis.

Head-box style respiration chambers were used to measure respiratory gas exchange for a single continuous 24 h for each block on d 16 between periods. Steers were placed into one of six (1 steer per chamber) positively pressurized ventilated hood-type REMS chambers. Features of the individual chambers included thermal environmental control to maintain animal comfort, fresh air supply for carbon dioxide (CO_2_) control, and measurement of incoming ventilation volumetric flow rate (Maia et al., 2015b). Lastly, gas sampling was conducted via a solenoid multiplexer to infrared photoacoustic gas analyzer (INNOVA 1412; LumaSense Technologies, Inc., Santa Clara, CA), configured with methane (CH_4_), ammonia (NH_3_), and sulfur hexafluoride (SF_6_) optical filters. More information regarding the REMS, including system description, operation, sampling integration time, and detection limits of the optical filters are reported by Maia et al. (2015a) and Ramirez et al. (2014). The last 5 of 10 gas concentration and thermal environment measurements at each sampling location (6 chambers and barn [incoming]) were averaged every 86 min for approximately 24 h. Before and after each period the steers were in the REMS, a mass recovery test was performed to verify mass measurement was within the expected range for these chambers. Methane emission rates were normalized to 24 h following a trapezoidal integration of the computed ER, resulting in a single CH_4_ emission (g/d) for each steer within each period. Methane and ammonia emissions were also expressed relative to DMI during the 24 h gas collection, DMI during the 5 d prior emission collection, and the digested DM and NDF using the prior 5-d DMI and the digestion coefficients that preceded the emission measurements. Feed and water were provided inside the chamber for ad libitum intake.

#### Laboratory analysis.

Feed, orts, and fecal samples were composited within collection period and lyophilized (FreeZone, Labconco, Kansas City, MO) and ground using a Wiley mill (1-mm screen, Arthur H. Thomas, Philadelphia, PA). Ground feed samples, orts, and feces were analyzed for DM (24 h at 105°C), NDF (Ankom200 Fiber Analyzer, Ankom Technology, Macedon, NY) using the Ankom method 5, nitrogen by combustion (Leco TruMac, LECO Corporation, St. Joseph, MI), fat (Ankom Technology, Macedon, NY) using the Ankom method 2, and total ash (12 h at 500°C; HotPack Muffle Oven 770750, HotPack Corp., Philadelphia, PA). Urine was analyzed for total nitrogen using the same nitrogen by combustion procedure as feed sample and urea content using a Beckman Coulter AU analyzer (Beckman Coulter, Brea, CA) at the University of Illinois Veterinary Diagnostic Laboratory. Results for total nitrogen analysis were deemed acceptable at a coefficient of variation of ≤5% within duplicates of feed, orts, urine, and feces.

#### Slaughter and carcass characteristics.

On d 35, steers were transported to the University of Illinois abattoir and humanely slaughtered under USDA inspection. At approximately 24 h postmortem, carcasses were evaluated for 12th-rib backfat thickness, LM area, percent KPH, ribeye lean maturity score, and ribeye marbling score by trained personnel.

### Experiment 2: Ruminal and In Vitro Fermentation

An additional experiment was conducted to validate nutrient digestibility results with a 2 × 2 factorial arrangement of treatments in split–plot design. A contemporary group of heifers (*N* = 19) were fed the same diet as steers in experiment 1 (Table 1) and whole plot treatments (dietary inclusion) were top-dressed including a control without ractopamine hydrochloride (**CON**, *n* = 9) or 400 mg/heifer/d ractopamine hydrochloride (**RAC**, *n* = 10). Heifers were adapted to the diet for 21 d prior to rumen fluid collection. Immediately prior to feeding, rumen fluid was collected by stomach tube from CON- and RAC-fed heifers, strained through four layers of cheesecloth, flushed with CO_2_, sealed in individual bottles, and transported to the laboratory in a warmed, insulated container to maintain temperature and limit oxygen exposure. Saliva contamination was minimized by disposing the initial 100 mL of collected rumen fluid. Ten heifers were sampled on d 22 in run 1 while the remaining 9 heifers were sampled the following day for run 2 to minimize time from sample collection to in vitro inoculation. Individual rumen fluid samples from each CON- and RAC-fed heifers was buffered in a 1:2 ratio (rumen fluid: buffer) with McDougall’s artificial saliva (McDougall, 1948). The buffered rumen fluid inoculum (105 mL) was added to 125 mL bottles fitted with a rubber stopper and a one-way valve. Each bottle contained 2.1 g of the lyophilized, ground diet, with subplot treatment (in vitro inclusion) of no ractopamine hydrochloride (**IVCON**) added to the ground diet substrate or ractopamine hydrochloride added (**IVRAC**). Ractopamine was included at a concentration of 3.9 mg/L to align with previous research (Walker and Drouillard, 2010) and the 400 mg RAC dose fed to the heifers. An incubator (Thermo Scientific, Waltham, MA) was used to maintain the fermentation bottles at 39°C under anaerobic conditions for 24 h. The ground substrate consisted of the same mixture as the diet in Exp. 1. The effect of RAC feeding to heifers was evaluated in triplicate using blank bottles at 0 h to evaluate differences in ruminal characteristics over the 21 d adaptation period. The in vitro treatments (IVCON and IVRAC) were each evaluated in triplicate bottles at 24 h with blanks included for 0 h to correct for DM, volatile fatty acids (VFA), and ammonia contained in the ruminal fluid inoculum from each animal. At the end of 24 h, the pH of each bottle was determined using a benchtop pH meter (Accumet Basic AB15, Fisher Scientific, Hampton, NH) and fermentation was terminated by the addition of 6 N HCl. Samples were filtered and dried at 60°C for at least 24 h before allowed to cool in desiccators and then weighed to determine dry matter disappearance. Aliquots of acidified fluid were used to determine VFA and ammonia concentrations. Volatile fatty acids were determined according to Erwin et al. (1961) by gas chromatography (HP1850 series gas chromatography Hewlett-Packard, Wilmington, DE) on a glass column. Ammonia-N was determined by colorimetric procedures described by Broderick and Kang (1980) using a UV-visible spectrophotometer (Perkin-Elmer Model 2380, Waltham, MA).

### Statistical Analysis

Data from Exp. 1 were analyzed as a randomized, complete block design with fixed effects of treatment, time (collection period), their interaction and random effect of block. Data for one steer in the first period was partially removed as an outlier based on urinary nitrogen values as well as the associated variables such as absorbed and retained nitrogen. Experiment 2 was analyzed as a split–plot design with whole plot of heifer dietary treatment and the split-plot of in vitro substrate. Volatile fatty acid concentration and ammonia were measured at 0 h in blank bottles and included in the split–plot model as a covariate. Random effects for experiment 2 included run and the interaction between heifer treatment and run. Both experiments were analyzed using the MIXED procedure of SAS (v 9.4; SAS Institute Inc., Cary, NC). Treatment effects and interactions were considered significantly different at *P* ≤ 0.05 and trends were discussed at 0.05 < *P* ≤ 0.10.

## RESULTS

### Experiment 1

#### Nutrient digestibility.

Although RAC inclusion had no effect (*P* = 0.51) on DMI, RAC-fed steers had decreased fecal DM output (2.1 vs. 2.5 kg DM/d; *P* = 0.04) compared with CON-fed steers (Table 2). Moreover, RAC-fed steers had greater apparent total tract DM digestibility (77.1% vs. 73.5%; *P* < 0.01), and NDF digestibility (65.6% vs. 60.2%; *P* < 0.01) than CON-fed steers. Furthermore, RAC-fed steers also had greater (*P* < 0.01) OM digestibility than CON-fed steers. Reflecting the tendency for decreased nitrogen excretion, RAC-fed steers demonstrated greater apparent total tract nitrogen digestibility (72.4% vs. 69.4%; *P* = 0.01) than CON-fed steers.

**Table 2.** Effects of ractopamine hydrochloride on intake, digestion, and nitrogen balance of finishing steers in Exp. 1^*a*^

	CON		RAC		*P* ^*b*^			
Item	Per. 1	Per. 2	Per. 1	Per. 2	SEM	Trt^*a*^	Time^*b*^	Trt × time
DMI, kg/d	9.0	9.9	8.9	9.3	0.74	0.51	0.22	0.66
Fecal DM, kg/d	2.3	2.7	2.1	2.1	0.22	0.04	0.36	0.37
Urine output, kg/d	8.4	6.6	6.7	8.4	1.53	0.99	0.96	0.25
Total tract digestion, %								
DM	74.3	72.7	76.7	77.6	1.15	<0.01	0.76	0.27
OM	75.5	74.0	77.9	79.1	1.15	<0.01	0.88	0.28
N	68.2	70.6	70.7	74.0	1.97	0.01	0.01	0.65
NDF	58.9	61.5	62.3	69.0	1.93	<0.01	<0.01	0.21
Nitrogen								
N intake, g/d	189.3	214.7	187.9	202.1	16.55	0.52	0.08	0.61
N absorption, g/d^*c*^	134.4	152.0	132.6	149.5	13.58	0.76	0.02	0.96
N retention, g/d	73.4	84.8	74.4	89.1	18.24	0.78	0.18	0.86
% of N intake	35.6	39.1	39.1	43.9	6.85	0.28	0.27	0.86
% of N absorbed	52.0	55.4	55.2	59.4	8.87	0.52	0.50	0.94
Total N output, g/d^*d*^	123.5	129.9	113.4	113.1	8.49	0.10	0.71	0.67
Fecal N, g/d	59.9	62.7	55.3	52.6	4.19	0.10	0.99	0.53
Urinary N, g/d	60.4	67.2	58.2	60.4	7.22	0.53	0.53	0.76
Urea N, g/d	49.8	53.8	45.7	49.5	7.08	0.51	0.54	0.98
Urea N, % urine N	74.7	79.4	78.7	81.0	2.65	0.28	0.17	0.64

^*a*^Steers received a top-dress containing no ractopamine hydrochloride (**CON**, *n* = 6) or 400 mg/steer/d ractopamine hydrochloride (**RAC**, *n* = 6) for 35 d before slaughter. Two collection periods (Per.) were conducted on d 8–13 (Per. 1) and d 22–27 (Per. 2).

^*b*^Trt = effect of RAC inclusion in the diet. Time = effect of period.

^*c*^Calculated as Intake N (g/d) – Fecal N (g/d).

^*d*^Calculated as Fecal N (g/d) + Urinary N (g/d).

Sampling period had no effect (*P* ≥ 0.22), on DMI, fecal or urinary output, apparent total tract DM or OM digestibility. However, fiber (NDF and N) digestibility was greater during the second collection period (*P* ≤ 0.01) than the first collection period.

#### Nitrogen balance and nitrogen species.

No interaction (*P* ≥ 0.53) between treatment and time was observed for any nitrogen balance parameter evaluated (Table 2). Although RAC inclusion did not affect nitrogen intake (*P* = 0.52), there was a tendency for RAC-fed steers to excrete less total nitrogen as urine and feces (113.3 vs. 126.7 g/d; *P* = 0.10) than CON-fed steers. This was primarily due to a tendency for decreased fecal nitrogen output (53.9 vs. 61.3 g/d; *P* = 0.10) of RAC-fed steers compared with CON-fed steers. Nonetheless, RAC inclusion had no effect (*P* = 0.53) on urinary nitrogen output. There was no difference (*P* = 0.78) in nitrogen retention between RAC- and CON-fed steers, despite the difference in nitrogen excretion. No difference (*P* = 0.76) in absorbed nitrogen was observed between RAC- and CON-fed steers. A time effect was observed (*P* = 0.02) for N absorption with steers exhibiting greater absorption during the second sampling period than the first. This was likely due to the tendency (*P* = 0.08) for greater N intake in the second period. Nitrogen retention findings were unchanged when expressed as a percentage of nitrogen intake (*P* = 0.28) or as a percentage of absorbed nitrogen (*P* = 0.52) with no difference between RAC- and CON-fed steers.

Expressed both in g/d and as a percentage of total urine nitrogen, urea-nitrogen excretion was not affected by RAC inclusion (*P* ≥ 0.28) or sampling period (*P* ≥ 0.17). No interaction between RAC inclusion and sampling period was observed (*P* ≥ 0.64) for urinary urea-nitrogen excretion.

#### Gaseous emissions.

Ractopamine inclusion had no effect (*P* = 0.15) on methane emission via eructation when expressed in g/d (Table 3). Because changes in DMI affect methane production (Johnson and Johnson, 1995; Benchaar et al., 2001), emissions were also expressed in g/kg of DMI during the 24 h test period as well as g/kg of DMI averaged over the previous 5 d. No differences (*P* = 0.77) in CH_4_ emission were observed when calculated in g/kg of in-chamber DMI. However, when expressed as g/kg of 5 d average DMI, there was a tendency (*P* = 0.08) for RAC-fed steers to have greater CH_4_ production than CON-fed steers. This tendency for a difference in CH_4_ emission between CON- and RAC-fed steers was similarly reflected when adjusted for digested DM (*P* = 0.08). However, no differences in CH_4_ were observed when expressed as g/kg of digested NDF (*P* = 0.14).

**Table 3.** Effects of ractopamine hydrochloride on eructated gaseous emissions of finishing steers in Exp. 1

	Treatment^*a*^			
Item	CON	RAC	SEM	*P-value*
Methane, g/d	172.5	209.5	16.78	0.15
Methane, g/kg DMI during 24 h test	39.5	38.2	6.80	0.77
Methane, g/kg 5 d average DMI	19.8	24.5	2.22	0.08
Methane, g/kg 5 d average DM digested	26.6	31.9	2.66	0.08
Methane, g/kg 5 d average NDF digested	33.8	39.3	3.96	0.14
Ammonia, g/d	7.5	5.3	2.97	0.21
Ammonia, g/kg DMI during 24 h test	1.5	0.8	0.29	<0.01
Ammonia, g/kg 5 d average DMI	0.8	0.6	0.24	0.16
Ammonia, g/kg 5 d average DM digested	1.1	0.8	0.33	0.13
Ammonia, g/kg 5 d average NDF digested	1.4	0.9	0.38	0.09

^*a*^Steers received a top-dress containing no ractopamine hydrochloride (**CON**, *n* = 6) or 400 mg/steer/d ractopamine hydrochloride (**RAC**, *n* = 6) for 35 d before slaughter.

Ractopamine inclusion also had no effect (*P* = 0.21) on ammonia emissions through eructation when expressed in total g/d, although RAC-fed steers demonstrated numerically lesser NH_3_ production than CON-fed steers. This numerical trend was supported when expressed as a g/kg of in-chamber DMI basis, with RAC-fed steers emitting less (*P* < 0.01) NH_3_ through eructation than CON-fed steers. Removing the variability associated with DMI during the 24 h collection, when expressed in g/kg of 5 d average DMI, differences in NH_3_ eructation are reduced (*P* = 0.16). As above, when expressed as g NH_3_/kg of digested DM no difference (*P* = 0.13) were observed. Nonetheless, when corrected for digested NDF, a tendency for greater (*P* = 0.09) ammonia emission in CON-fed steers was observed.

#### Carcass characteristics.

No difference (*P* ≥ 0.16) in HCW, dressing %, 12th-rib fat thickness, or LM area of CON- and RAC-fed steers was observed (Supplementary Table S1). Despite a lack of statistical significance, the LM area of RAC-fed steers was 5.03 cm^2^ greater than CON-fed steers. Nonetheless, no differences (*P* ≥ 0.31) in calculated USDA yield grade was observed, an expected response given the lack of difference in 12th-rib fat thickness. There was a tendency (*P* = 0.06) for carcasses from RAC-fed steers to have greater KPH than those from CON-fed steers. From a meat quality standpoint, RAC usage had no effect (*P* ≥ 0.16) on the marbling score.

### Experiment 2

#### Ruminal and in vitro fermentation.

Dietary inclusion of RAC directly affected in vivo ruminal fermentation by decreasing (*P* < 0.01) ruminal pH collected 24 h after feeding in RAC-fed heifers compared with CON-fed heifers (7.04 vs. 7.16, respectively) and tending to increase (*P* = 0.06) total VFA concentration in RAC-fed heifers (Table 4). Furthermore, there was a tendency (*P* = 0.08) for a decreased molar percentage of propionate in RAC-fed heifers than CON-fed heifers prior to feeding. Dietary ractopamine inclusion did not affect (*P* ≥ 0.15) molar percentage of acetate or butyrate; however, a tendency (*P* = 0.07) for greater acetate:propionate ratio was observed in RAC-fed heifers compared with CON-fed heifers. No differences (*P* = 0.67) in in vivo ruminal NH_3_ were observed.

**Table 4.** Effects of ractopamine hydrochloride inclusion on ruminal fermentation in vivo^*a*^

	Treatment^*b*^			
Item	CON	RAC	SEM	*P-value*
pH	7.16	7.04	0.06	<0.01
Ammonia N, m*M*	0.90	1.02	0.20	0.67
Total VFA, m*M*	75.84	90.30	2.64	0.06
VFA, mol/100 mol				
Acetate	45.34	48.05	1.31	0.15
Propionate	40.31	33.82	2.55	0.08
Butyrate	9.98	13.43	1.55	0.13
Isobutyrate	1.07	1.34	0.27	0.18
Valerate	1.45	1.43	0.08	0.84
Isovalerate	1.83	1.93	0.18	0.68
Acetate:propionate	1.16	1.54	0.14	0.07

^*a*^Rumen fluid samples were collected by stomach tube from CON- and RAC-fed heifers prior to feeding.

^*b*^Heifers received a dietary top-dress containing no ractopamine hydrochloride (**CON**, *n* = 9) or 400 mg/heifer/d ractopamine hydrochloride (**RAC**, *n* = 10).

In vitro fermentation after 24 h incubation of the same dietary substrate was also influenced by both dietary and in vitro ractopamine inclusion (Table 5). A dietary RAC × in vitro RAC interaction (*P* = 0.05) was detected for final pH. Although in vitro pH was decreased in bottles containing the IVRAC substrate compared with IVCON substrate regardless of inoculation with rumen fluid of CON- or RAC-fed heifers, a greater magnitude of decrease between IVCON and IVRAC bottles was observed when inoculated with rumen fluid from RAC-fed heifers. No other interactions (*P* ≥ 0.16) between dietary and in vitro ractopamine inclusion were observed. In vitro inclusion of ractopamine affected NH_3_ production with decreased (*P* < 0.01; 4.73 vs. 6.11 mM) NH_3_ observed in bottles containing the IVRAC substrate than those containing the IVCON substrate. Although neither dietary nor in vitro ractopamine inclusion affected (*P* ≥ 0.31) total VFA concentration, differences in VFA molar percentages were observed. Similar to in vivo results, a tendency was observed for bottles inoculated with rumen fluid of RAC-fed heifers to exhibit a decreased (*P* = 0.10) molar percentage of propionate and increased (*P* = 0.10) acetate:propionate ratio compared with bottles inoculated with rumen fluid of RAC-fed heifers. However, whereas in vitro inclusion of ractopamine had no effect (*P* ≥ 0.33) on molar percentages of acetate or propionate, a slightly greater (15.18% vs. 14.75%; *P* = 0.04) molar percentage of butyrate was observed in bottles containing the IVRAC substrate than in those with IVCON substrate. Decreased (*P* < 0.01) molar percentages of both valerate and isovalerate were observed in bottles containing the IVRAC substrate compared with in those containing IVCON substrate.

**Table 5.** Effects of ractopamine hydrochloride inclusion on in vitro fermentation after 24 h incubation in Exp. 2^*^

	Dietary CON		Dietary RAC			*P-value*		
	IVCON	IVRAC	IVCON	IVRAC	SEM	Diet	INV	D × INV
pH^†^	6.20^a^	6.05^c^	6.15^bc^	5.91^d^	0.03	0.04	<0.01	0.05
Ammonia N, mM	6.03	4.72	6.19	4.72	2.19	0.98	<0.01	0.40
Total VFA, mM	78.06	78.24	80.06	81.25	1.75	0.31	0.44	0.56
Molar percentage								
Acetate	43.01	42.60	42.27	42.07	0.67	0.49	0.33	0.75
Propionate	37.13	37.45	36.22	35.62	0.57	0.10	0.66	0.16
Butyrate	14.43	14.80	15.08	15.57	0.90	0.58	0.04	0.76
Isobutyrate	1.10	1.12	1.29	1.27	0.08	0.14	0.96	0.32
Valerate	2.65	2.52	2.98	2.83	0.15	0.12	<0.01	0.75
Isovalerate	2.08	1.91	2.47	2.30	0.17	0.06	<0.01	0.99
Acetate:propionate	1.16	1.14	1.18	1.21	0.02	0.10	0.86	0.26

^*^Heifers received no ractopamine hydrochloride (**CON**, *n* = 9) or 400 mg·heifer-1·d-1 ractopamine hydrochloride (**RAC**, *n* = 10). Rumen fluid samples were collected by stomach tube from CON- and RAC-fed heifers prior to feeding. In vitro treatments including negative control (**IVCON**) and ractopamine hydrochloride (**IVRAC**) were added to the in vitro substrate.

Diet = the effect of dietary inclusion of RAC. INV = the effect of RAC inclusion in the in vitro substrate. D × INV = interaction between dietary RAC inclusion and in vitro RAC inclusion.

^†^Treatments lacking common superscripts (a-d) differ (*P* ≤ 0.05).

**Figure 1.**
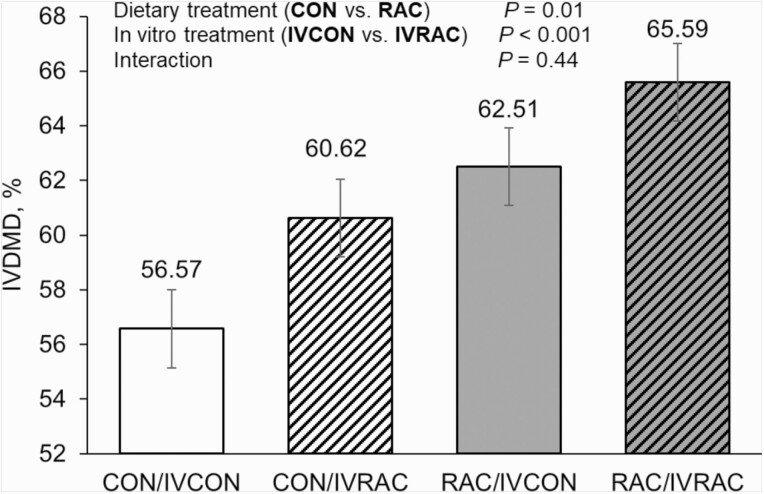
Effects of dietary and in vitro inclusion of ractopamine hydrochloride on in vitro dry matter disappearance (**IVDMD**) of finishing beef heifers Exp. 2. White bars correspond to heifers that received no ractopamine hydrochloride (**CON**, *n* = 9) and dark bars correspond to heifers that received 400 mg·heifer-1·d-1 ractopamine hydrochloride (**RAC**, *n* = 10). Bars without stripes correspond to the in vitro negative control (**IVCON**) treatment containing no ractopamine and bars with stripes correspond to the in vitro ractopamine hydrochloride treatment (**IVRAC**) which was added to the substrate.

Dietary inclusion of RAC affected IVDMD with greater (64.0% vs. 58.6%; *P* = 0.01) DM disappearance observed in bottles containing rumen fluid from RAC-fed heifers than bottles containing rumen fluid from CON-fed heifers (Figure 1). In vitro inclusion of ractopamine also affected IVDMD with greater (63.1% vs. 59.5%; *P* < 0.01) DM disappearance observed in bottles containing the IVRAC substrate than in those with IVCON substrate. Despite significant main effects, no interaction (*P* = 0.44) between dietary ractopamine inclusion and in vitro ractopamine inclusion was observed.

## DISCUSSION

Recent consumer and policy-maker interest in environmental sustainability has spurred new interest in developing methods to decrease the environmental footprint of multiple industries, including beef production. On average, only 10–20% of fed nitrogen is retained in animal tissue with excess nutrient excretion not only reducing profits through forfeited feed costs, but also creating environmental challenges as well. Formation and volatilization of the nitrogenous molecules NH_3_ and N_2_O are major contributors to air, soil, and water pollution in both terrestrial and aquatic ecosystems contributing to increased aerosol formation, soil acidity, and eutrophication (Hristov et al., 2011).

Considering 57–67% of the total excreted nitrogen is lost to volatilization (Bierman et al., 1999), excess excretion has serious environmental implications. In Exp. 1, RAC feeding resulted in a 10.6% decrease in nitrogen excretion. Others have reported similar findings for nitrogen excretion regardless of ractopamine dosage. Although N excretion was not different, Carmichael et al. (2018) observed a 10.9% numerical decrease in nitrogen excretion of steers fed 300 mg/steer/d RAC for 28 d and Walker et al. (2007) observed a 10.8% decrease in steers fed 200 mg/steer/d^-^ RAC. Data have revealed little difference in nitrogen absorption of RAC-fed steers (Walker et al., 2007; Carmichael et al., 2018), indicating a limited likelihood of absorption-linked effects. Nonetheless, in Exp.1 a time effect was observed for greater N absorption. However, changes in N absorption over time are likely tied to a tendency for slightly greater N intake in sampling period two than one.

Effects of β-AA use on urea kinetics and total *N* output are less well defined in the literature. Representing 60–90% of total urinary nitrogen excreted in cattle (Bristow et al., 1992), urea nitrogen is highly susceptible to volatilization as NH_3_ given the abundance of ureolytic compounds present in manure and soil (Hristov et al., 2011). Cole et al. (2005) demonstrated urinary N excretion was highly correlated (*r* = 0.83) to environmental ammonia losses. Although no decrease in urea nitrogen was observed in Exp. 1, Walker et al. (2007) reported reduced urea nitrogen in urine of RAC-fed steers. However, when standardized for differences in nitrogen intake between treatments, urea nitrogen effects were considerably diminished. Nonetheless, a study evaluating the effects of β-AA on urea recycling reported a tendency for decreased urea-nitrogen entry rate and urea-nitrogen recycled to the gastrointestinal tract in zilpaterol-fed steers when adjusted for nitrogen intake (Brake et al., 2011). Given these findings and the lack of nitrogen absorption effects observed between treatments, data suggest nitrogen repartitioning effects observed in RAC-fed steers may be a result of reduced urea production and recycling.

Steers fed RAC in Exp. 1 exhibited less pronounced effects on nitrogen retention compared with earlier reports of 14% and 28% increases in nitrogen retention reported by Carmichael et al. (2018) and Walker et al. (2007), respectively. Although carcass effects traditionally associated with the use of ractopamine were not observed in Exp. 1 due to the smaller number of animals used in metabolism trials, numerical improvements in the LM area were observed suggesting greater nitrogen incorporation through increased protein accretion. This assumption is supported when considering the observed 2.6 g daily increase in nitrogen retention over a 35 d period approximately equates to an additional 0.5 kg of carcass protein accretion (assuming a nitrogen conversion factor of 6.25).

Walker and Drouillard (2010) revealed grain processing methods, used to improve starch utilization of feed ingredients, may be another important consideration when evaluating nitrogen and urea kinetics in RAC-fed finishing steers. An in vivo experiment reported ruminal ammonia concentrations were more affected by RAC supplementation in diets containing dry rolled corn than in diets containing steam-flaked corn (Walker and Drouillard, 2010). Although ruminal ammonia concentrations were not evaluated in Exp. 1, decreased ruminal ammonia concentrations associated with RAC usage when feeding DRC, as observed by Walker and Drouillard, (2010), suggest greater bypass protein or microbial crude protein availability.

In Exp. 1, feeding RAC resulted in an improvement in nutrient digestibility. However, of the relatively few studies evaluating the effects of feeding β-AAs on nutrient digestibility and ruminal fermentation, mixed results have been observed. Several studies in lambs have shown no difference in DM digestion when fed cimaterol (10 mg/kg; Kim et al., 1989) or RAC (López-Carlos et al., 2010) compared with control-fed lambs. In beef, Strydom et al. (2009) reported steers fed RAC (30 mg/kg) exhibited DM and CP digestion similar to control-fed steers. More similar to the findings of Exp. 1, Walker et al. (2007) reported a 2% improvement in DM digestibility of RAC-fed steers fed 200 mg/steer/d RAC over control-fed steers.

Improvements in nutrient digestion related to β-AAs have been hypothesized to be a response to the binding of beta-adrenergic receptors present in the gut, inhibiting ruminal contractions, resulting in greater retention time and greater ultimate digestion (Brikas, 1989).

An alternative hypothesis for the mechanism behind greater nutrient digestibility of RAC-fed steers is that RAC usage may have a direct effect on non-mammalian cells. Previous in vitro research has confirmed the stimulatory effects of catecholamines on bacterial species. Both Lyte and Ernst (1992) and Kinney et al. (2000) demonstrated catecholamines dramatically increased gram-negative bacterial species. Results of a series of in vivo studies evaluating the effects of RAC usage on foodborne pathogens in lambs, pigs, and feedlot cattle indicated RAC may affect gut microflora populations. Edrington et al. (2006a) demonstrated increased *Escherichia coli* O157:H7 fecal shedding in experimentally inoculated lambs administered RAC and decreased *Salmonella* shedding in inoculated pigs administered RAC. In a separate experiment, Edrington et al. (2006b) reported decreased *E. coli* O157:H7 shedding in inoculated feedlot cattle fed RAC, but increased *Salmonella* shedding. A series of in vitro experiments by Walker and Drouillard (2010) provided further evidence for the effects of RAC on ruminal fermentation. Similar to results of Exp. 2, Walker and Drouillard (2010) demonstrated greater IVDMD of the ground diet included in bottles inoculated with rumen fluid containing RAC. However, given the additive effects of dietary and in vitro RAC inclusion in Exp. 2 (Figure 1), it appears improvements in IVDMD may be the result of different mechanisms. Ungemach (2004) reported peak blood plasma concentration for ractopamine at 0.5–2 h after dosing and elimination half-life at 6–7 h after initial dosing. With rumen fluid having been collected prior to feeding, significant effects observed in the whole plot (heifer) would possibly be the result of long-term selection pressure on the microbial population such that microbiota present in rumen fluid of RAC-fed heifers better digested the diet substrate.

Effects of β-AAs on nutrient digestibility may also be somewhat dosage dependent. Walker and Drouillard (2010) observed both greater IVDMD and gas production with increasing RAC concentrations, up to a certain point, lending credence to a greater increase in DM digestibility observed in Exp. 1 (400 mg/steer/d) compared with the Walker et al. (2007) study (200 mg/steer/d). Given the increase in fermentative gas production with greater RAC concentrations observed by Walker and Drouillard (2010), it is possible changes in ruminal fermentation may slightly increase the amount of eructated methane in vivo. The theory is further supported by the absence of a concomitant increase in VFA production observed by Walker and Drouillard (2010) suggesting methanogen species may be responsible for increased gas production.

Although no differences in total eructated methane and ammonia were observed between control and RAC-fed cattle, methane emissions in beef cattle are dependent on several factors including animal size, dietary composition, and feed intake (Johnson and Johnson, 1995; Beuchemin and McGinn, 2006). As individual feed intake differences were observed during the gaseous emission test, data were corrected for DMI during the 24 h test period. On comparison to other respiration calorimetry literature evaluating eructated methane (Hales et al., 2012), elevated heat production values and depressed respiratory quotient values (data not shown) observed in Exp. 1. indicated steers were moderately uncomfortable during the 24 h test period. For this reason, authors chose to also standardize methane and ammonia emission data for DMI of the 5 d prior to the emission test. Furthermore, given RAC-fed steers exhibited greater NDF digestibility, CH_4_ and NH_3_ production were also expressed as g/kg of NDF digested. Matching total methane production results, no difference in methane standardized for 5-d average digested DM and NDF was observed between CON- and RAC-fed steers.

Results of Exp. 1. indicated feeding RAC resulted in a 10.6% reduction in total nitrogen excretion over the 35 d feeding period. Despite minor differences in nutrient digestibility and N absorption over time, overall data does not suggest β-AA receptor desensitization were observed after feeding RAC for 27 d. Improvements in total tract nutrient digestion of RAC-fed steers were unexpected given previous research. However, these findings were supported by Exp. 2. results demonstrating greater IVDMD in bottles incubated with rumen fluid from RAC-fed heifers and bottles containing a substrate with RAC. These findings indicate ractopamine inclusion can impact microbial digestion of a dry-rolled corn-based finishing diet and reduce N output in feedlot cattle.

## Supplementary Material

txab036_suppl_Supplementary_Materials

## References

[CIT0001] Beauchemin, K. A. and S. M. McGinn. 2006. Enteric methane emissions from growing beef cattle as affected by diet and level of intake. Can. J. Anim. Sci. 86:401–408. doi:10.4141/A06-021

[CIT0002] Benchaar, C., C. Pomar, and J. Chiquette. 2001. Evaluation of dietary strategies to reduce methane production in ruminants: a modeling approach. Can. J. Anim. Sci. 81:563–574 doi:10.4141/A00-119

[CIT0003] Bierman, S., G. E. Erickson, T. J. Klopfenstein, R. A. Stock, and D. H. Shain. 1999. Evaluation of nitrogen and organic matter balance in the feedlot as affected by level and source of dietary fiber. J. Anim. Sci. 77:1645–1653. doi:10.2527/1999.7771645x 10438008

[CIT0004] Boler, D. D., S. F. Holmer, F. K. McKeith, J. Killefer, D. L. VanOverbeke, G. G. Hilton, R. J. Delmore, J. L. Beckett, J. C. Brooks, R. K. Miller, et al. 2009. Effects of feeding zilpaterol hydrochloride for twenty to forty days on carcass cutability and subprimal yield of calf-fed Holstein steers. J. Anim. Sci. 87:3722–3729. doi:10.2527/jas.2009-1830 19574574

[CIT0005] Brake, D. W., E. C. Titgemeyer, and M. L. Jones. 2011. Effect of nitrogen supplementation and zilpaterol–HCl on urea kinetics in steers consuming corn-based diets. J. Anim. Physiol. Anim. Nutr. 95:409–416. doi:10.1111/j.1439-0396.2010.01064.x 21039927

[CIT0006] Brikas, P. 1989. The adrenergic receptors in the control of reticulo-rumen myoelectrical activity in sheep. J. Vet. Med. A. 36:402–410. doi:10.1111/j.1439-0442.1989.tb00747.x 2508369

[CIT0007] Bristow, A. W., D. C. Whitehead, and J. E. Cockburn. 1992. Nitrogenous constituents in the urine of cattle, sheep and goats. J. Sci. Food Agric. 59:387–394. doi:10.1002/jsfa.2740590316

[CIT0008] Broderick, G. A., and J. H. Kang. 1980. Automated simultaneous determination of ammonia and total amino acids in ruminal fluid and in vitro media. J. Dairy Sci. 63:64–75. doi:10.3168/jds.S0022-0302(80)82888-8 7372898

[CIT0009] Capper, J. L. 2011. The environmental impact of beef production in the United States: 1977 compared with 2007. J. Anim. Sci. 89:4249–4261. doi:10.2527/jas.2010-3784 21803973

[CIT0010] Capper, J. L., and D. J. Hayes. 2012. The environmental and economic impacts of removing growth-enhancing technologies from U.S. beef production. J. Anim. Sci. 90:3527–3537. doi:10.2527/jas.2011-4870 22665660

[CIT0011] Carmichael, R. N., O. N. Genther-Schroeder, C. P. Blank, E. L. Deters, S. J. Hartman, E. K. Niedermayer, and S. L. Hansen. 2018. The inﬂuence of supplemental zinc and ractopamine hydrochloride on trace mineral and nitrogen retention of beef steers. J. Anim. Sci. 96:2939–2948. doi:10.1093/jas/sky177 29733402 PMC6095283

[CIT0012] Cole, N. A., R. N. Clark, R. W. Todd, C. R. Richardson, A. Gueye, L. W. Greene, and K. McBride. 2005. Influence of dietary crude protein concentration and source on potential ammonia emissions from beef cattle manure. J. Anim. Sci. 83:722–731. doi:10.2527/2005.833722x 15705770

[CIT0013] Cooprider, K. L., F. M. Mitloehner, T. R. Famula, E. Kebreab, Y. Zhao, and A. L. Van Eenennaam. 2011. Feedlot efficiency implications on greenhouse gas emissions and sustainability. J. Anim. Sci. 89:2643–2656. doi:10.2527/jas.2010-3539 21398565

[CIT0014] Edrington, T. S., T. R. Callaway, S. E. Ives, M. J. Engler, T. H. Welsh, D. M. Hallford, K. J. Genovese, R. C. Anderson, and D. J. Nisbet. 2006a. Effect of ractopamine HCl supplementation on *Escherichia coli* O157:H7 and *Salmonella* in feedlot cattle. Curr. Micro. 53:340–345. doi:10.1007/s00284-006-0200-9 16972129

[CIT0015] Edrington, T. S., T. R. Callaway, D. J. Smith, K. J. Genovese, R. C. Anderson, and D.J. Nisbet. 2006b. Effect of ractopamine HCl on *Escherichia coli* O157:H7 and *Salmonella* in vitro and on intestinal populations and fecal shedding in experimentally infected sheep and pigs. Curr. Micro. 53:82–88. doi:10.1007/s00284-006-0019-4 16775793

[CIT0016] Erwin, E. S., G. J. Marco, and E. M. Emery. 1961. Volatile fatty acid analyses of blood and rumen fluid by gas chromatography. J. Dairy Sci. 44:1768–1771. doi:10.3168/jds.S0022-0302(61)89956-6

[CIT0016a] FASS. 2010. Guide for the care and use of agricultural animals in agricultural research and teaching: consortium for developing a guide for the care and use of agricultural research and teaching. Champaign (IL): FASS Association Headquarters.

[CIT0017] Hales, K. E., N. A. Cole, and J. C. MacDonald. 2012. Effects of corn processing method and dietary inclusion of wet distillers grains with solubles on energy metabolism, carbon–nitrogen balance, and methane emissions of cattle. J. Anim. Sci. 90:3174–3185. doi:10.2527/jas.2011-4441 22585790

[CIT0018] Hausdorff, W. P., M. G. Caron, and R. J. Lefkowitz. 1990. Turning off the signal: desensitization of beta-adrenergic receptor function. Faseb J. 4:2881–2889. 2165947

[CIT0019] Hilton, G. G., J. L. Montgomery, C. R. Krehbiel, D. A. Yates, J. P. Hutcheson, W. T. Nichols, M. N. Streeter, J. R. Blanton Jr., and M. F. Miller. 2009. Effects of feeding zilpaterol hydrochloride with and without monensin and tylosin on carcass cutability and meat palatability of beef steers. J. Anim. Sci. 87:1394–1406. doi:10.2527/jas.2008-1170. 19028853

[CIT0020] Hristov, A. N., M. Hanigan, A. Cole, R. Todd, T. A. McAllister, P. M. Ndegwa, and A. Rotz. 2011. Review: ammonia emissions from dairy farms and beef feedlots. Can. J. Anim. Sci. 91:1–35. doi:10.4141/CJAS10034

[CIT0021] Johnson, K. A., and D. E. Johnson. 1995. Methane emissions from cattle. J. Anim. Sci. 73:2483–2492. doi:10.2527/1995.7382483x 8567486

[CIT0022] Kim, Y. S., Y. B. Lee, W. N. Garrett, and R. H. Dalrymple. 1989. Effects of cimaterol on nitrogen retention and energy utilization in lambs. J. Anim. Sci. 67:674–681. doi:10.2527/jas1989.673674x 2566593

[CIT0023] Kinney, K. S., C. E. Austin, D. S. Morton, and G. Sonnenfeld. 2000. Norepinephrine as a growth stimulating factor in bacteria–mechanistic studies. Life Sci. 67:3075–3085. doi:10.1016/s0024-3205(00)00891-2 11125844

[CIT0024] Lopez-Carlos, M. A., R. G. Ramírez, J. I. Aguilera-Soto, C. F. Aréchiga, F. Méndez-Llorente, H. Rodríguez, and J. M. Silva. 2010. Effect of ractopamine hydrochloride and zilpaterol hydrochloride on growth, diet digestibility, intake, and carcass characteristics of feedlot lambs. Liv. Sci. 131:23–30 doi:10.1016/j.livsci.2010.02.018

[CIT0025] Lyte, M., and S. Ernst. 1992. Catecholamine induced growth of gram negative bacteria. Life Sci. 50:203–212. doi:10.1016/0024-3205(92)90273-r 1731173

[CIT0026] Maia, G. D. N., B. C. Ramirez, A. R. Green, L. F. Rodriguez, J. R. Segers, D. W. Shike, and R. S. Gates. 2015a. A novel ruminant emission measurement system: Part I. Design evaluation and description. Trans. ASABE. 58(3): 749–762. doi:10.13031/trans.58.10752

[CIT0027] Maia, G. D. N., B. C. Ramirez, A. R. Green, L. F. Rodriguez, J. R. Segers, D. W. Shike, and R. S. Gates. 2015b. A novel ruminant emission measurement system: Part II. Commissioning. Trans. ASABE. 58(6): 1801–1815. doi:10.13031/trans.58.10753

[CIT0028] McDougall, E. I. 1948. Studies on ruminant saliva. 1. The composition and output of sheep’s saliva. Biochem. J. 43: 99–109. doi:10.1042/bj0430099 PMC1274641 16748377

[CIT0029] NASEM. 2016. Nutrient requirements of beef cattle. 8th Revised ed. Washington (DC): National Academies Press.

[CIT0030] Prados, L. F., M. L. Chizzotti, S. C. V. Filho, F. H. M. Chizzotti, P. P. Rotta, and L. F. C. Silva. 2016. Environmental management and prediction of nitrogen and phosphorus excretion by beef cattle. Nutrient Requirements of Zebu and Crossbred Cattle (BR-CORTE). 3rd ed. S.C.V. Filho and L.F.C. Silva. (Ed.) Brazil: Suprema Gráfica Ltda.

[CIT0031] Ramirez, B. C., G. D. N. Maia, A. R. Green, D. W. Shike, L. F. Rodríguez, and R. S. Gates. 2014. Design and validation of a precision orifice meter for ventilation rate control in open-circuit respiration chambers. Trans. ASABE 57(6):1865–1872. doi:10.13031/trans.57.10754

[CIT0032] Schröder, J., A. Bannink, and R. Kohn. 2005. Improving the efficiency of nutrient use on cattle operations. In: E. Pfeffer and A.N. Hristov, editors, Nitrogen and phosphorus nutrition of cattle: reducing the environmental impact of cattle operations. Cambridge, MA: CABI Publishing. p. 255–279.

[CIT0033] Strydom, P. E., L. Frylinck, J. L. Montgomery, and M. F. Smith. 2009. The comparison of three β-agonists for growth performance, carcass characteristics and meat quality of feedlot cattle. Meat Sci. 81:557–564. doi:10.1016/j.meatsci.2008.10.011 20416591

[CIT0034] Ungemach, F. R. 2004. Ractopamine (addendum). WHO Food Additive Series: 53 – [accessed October 23, 2017]. http://www.inchem.org/documents/jecfa/jecmono/v53je08.htm.

[CIT0035] Walker, C. E., and J. S. Drouillard. 2010. Effects of ractopamine hydrochloride are not confined to mammalian tissue: evidence for direct effects of ractopamine hydrochloride supplementation on fermentation by ruminal microorganisms. J. Anim. Sci. 88:697–706. doi:10.2527/jas.2009-1999 19820054

[CIT0036] Walker, D. K., E. C. Titgemeyer, E. K. Sissom, K. R. Brown, J. J. Higgins, G. A. Andrews, and B. J. Johnson. 2007. Effects of steroidal implantation and ractopamine-HCl on nitrogen retention, blood metabolites, and skeletal muscle gene expression in Holstein steers. J. Anim. Physiol. Anim. Nutr. 91: 439–447. doi:10.1111/j.1439-0396.2007.00675.x 17845252

