# Supplementary material for: Effects of ractopamine hydrochloride on nutrient digestibility and nitrogen excretion of finishing beef cattle
Source: Transl Anim Sci. 2021 Mar 7;5(2):txab036. doi: 10.1093/tas/txab036 (PMC8628867; doi:10.1093/tas/txab036)
Supplement: txab036_suppl_Supplementary_Materials [file txab036_suppl_Supplementary_Materials.docx]

## **Supplemental Material**

## **Effects of ractopamine hydrochloride on nutrient digestibility and nitrogen excretion of finishing beef cattle**

*B. N. Harsh^1^, B. J. Klatt, M. J. Volk, A. R. Green-Miller, J. C. McCann*^2^

University of Illinois at Urbana-Champaign, IL 61801, USA

| **Table S1.** Effects of ractopamine hydrochloride on carcass characteristics and meat quality traits of finishing steers in Exp. 1 | | | | |
| --- | --- | --- | --- | --- |
|  | Treatment^1^ | |  |  |
| Item | CON | RAC | SEM | *P*-value |
| Final BW, kg | 571 | 572 | 9.0 | 0.93 |
| Carcass characteristics |  |  |  |  |
| HCW, kg | 353 | 354 | 5.4 | 0.96 |
| Dressing % | 64.7 | 64.3 | 0.9 | 0.24 |
| LM area, cm^2^ | 79.2 | 84.2 | 2.3 | 0.16 |
| 12^th^-rib fat thickness, cm | 1.25 | 1.29 | 0.12 | 0.82 |
| KPH fat, % | 2.3 | 2.6 | 0.14 | 0.06 |
| USDA yield grade^2^ | 3.12 | 2.95 | 0.20 | 0.57 |
| Marbling score^3^ | 445 | 452 | 15.6 | 0.77 |
| ^1^ Steers received a top-dress containing no ractopamine hydrochloride (**CON**, n = 6) or 400 mg/steer/d ractopamine hydrochloride (**RAC**, n = 6) for 35 d before slaughter | | | | |
| ^2^ Calculated USDA yield grade = (2.5+ [2.5*12^th^-rib fat thickness] +[0.2*KPH%] +[0.0038*HCW]- [0.32*LM area]) | | | | |
| ^3^ Marbling scores: 400 = Small^00^ (low choice) and 500 = Modest^00^ (average choice) | | | | |
